# HIV-1 Tat protein vaccination in mice infected with *Mycobacterium tuberculosis* is safe, immunogenic and reduces bacterial lung pathology

**DOI:** 10.1186/s12879-016-1724-7

**Published:** 2016-08-22

**Authors:** Aurelio Cafaro, Giovanni Piccaro, Giuseppe Altavilla, Vincenzo Gigantino, Giuseppe Matarese, Erika Olivieri, Flavia Ferrantelli, Barbara Ensoli, Carla Palma

**Affiliations:** 1 National AIDS Center, Istituto Superiore di Sanità, Viale Regina Elena, Rome, 299 00161 Italy; 2 Department of Infectious, Parasitic and Immune-mediated Diseases, Istituto Superiore di Sanità, Viale Regina Elena, Rome, 299 00161 Italy; 3 Institute of Pathology, University of Padua, Padua, Italy; 4 Istituto di Endocrinologia e Oncologia Sperimentale, Consiglio Nazionale delle Ricerche (IEOS-CNR) c/o Dipartimento di Medicina Molecolare e Biotecnologie Mediche, Università di Napoli Federico II, Naples, Italy; 5 Dipartimento di Patologia Fondazione G. Pascale IRCCS, Naples, Italy; 6 Dipartimento di Medicina e Chirurgia, Università di Salerno, Salerno, Italy; 7 MultiMedica IRCCS, Milan, Italy

**Keywords:** Tat vaccination, *M. tuberculosis* infection, Cytokines, T cell responses, Antibodies, Rodent

## Abstract

**Background:**

The therapeutic HIV-1 Tat protein vaccine is in advanced clinical development. Tuberculosis, the main AIDS co-infection, is highly endemic in areas where AIDS prevention through vaccination is needed. However, safety and immunogenicity of Tat vaccination in the course of *Mycobacterium tuberculosis (Mtb)* infection is still unknown and it prevents the possibility to administer the vaccine to *Mtb*-infected individuals. We addressed the interplay and effects of Tat vaccination on *Mtb* infection in immunocompetent mice.

**Methods:**

C57BL/6 mice were vaccinated or not with Bacillus Calmette-Guerin (BCG), the current tuberculosis vaccine, and after 5 weeks were infected with *Mtb* by intravenous route*.* The Tat protein was injected intradermally at 1, 2 and 4 weeks after *Mtb* challenge. Eight weeks after *Mtb* infection, all mice were sacrificed, and both the degree of pathology and immune responses to *Mtb* and Tat were evaluated. As additional control, some mice were either vaccinated or not with BCG, were not challenged with *Mtb,* but received the Tat protein. Statistical significances were evaluated by one-way or two-way ANOVA and Tukey’s multiple comparisons post-test.

**Results:**

In the lungs of *Mtb*-infected mice, Tat-vaccine did not favour *Mtb* replication and indeed reduced both area of cellular infiltration and protein levels of Interferon-γ, Chemokine (C-C motif) ligand-4 and Interleukin-1β, pathological events triggered by *Mtb*-infection. Moreover, the protection against *Mtb* infection conferred by BCG remained good after Tat protein treatment. In spleen cells of *Mtb*-infected mice, Tat vaccination enhanced *Mtb*-specific Interferon-γ and Interleukin-17 responses, which may have a protective role. Of note, *Mtb* infection reduced, but did not suppress, the development of anti-Tat antibodies, required for Tat vaccine efficacy and the titer of anti-Tat IgG was potentiated by BCG vaccination in *Mtb*-free mice. In general, Tat treatment was well tolerated in both *Mtb*-infected and *Mtb*-free mice.

**Conclusions:**

Tat protein vaccine, administered in *Mtb*-infected mice with a protocol resembling that used in the clinical trials, was safe, immunogenic, limited the lung *Mtb*-associated immunopathology and did not abrogate the protective efficacy of BCG. These data provide preliminary evidence for a safe use of Tat vaccine in people vaccinated with BCG and/or suffering from tuberculosis.

## Background

The HIV regulatory Tat protein is crucial in AIDS pathogenesis and is a promising vaccine candidate in advanced clinical development. Tat is the transactivator of HIV gene expression and it is essential for viral replication, establishment of infection and virus reactivation [1, 2]. Tat is expressed by proviral DNA prior to virus integration into the host genome [3], and it is commonly found extracellularly both during acute infection and at the time of virus reactivation [4, 5], even in patients on effective antiretroviral therapy [6]. Extracellular Tat protein concurs to cell-to-cell virus transmission, disease progression [4, 7] and immune dysregulation [8], contributing to the chronic immune hyperactivation and dysfunction observed in HIV infection [3, 9].

Approaches employing biologically active Tat protein have been shown to contain virus replication, preventing disease onset and/or progression in monkey models [10, 11], (http://www.hiv1tat-vaccines.info). The Tat-based vaccine has then been advanced to clinical testing in preventative phase I, and therapeutic phase I and II trials showing safety and immunogenicity [12–17]. Moreover, two different trials indicated that Tat vaccine contributed to HIV-1 containment in patients on effective HAART [14, 18], (ISS T-003, *ClinicalTrial.gov identifier: NCT01513135*). In particular, results from an open-label randomized exploratory therapeutic phase II trial in 168 patients on effective HAART showed that vaccination induced a durable and significant restoration of T, B, natural killer cells, and CD4^+^ and CD8^+^ central memory subsets, as well as up-regulation of the expression of HLA-DR^+^ on CD8^+^ killer T cells, a phenotype found to be increased in elite controllers [16] and to contribute to HIV-1 containment [17]. Moreover, the Tat vaccine induced a significant reduction of blood proviral DNA which was significantly associated with anti-Tat immunoglobulin (Ig)M and IgG antibody titers and neutralization of Tat-mediated entry of oligomeric Env in dendritic cells [14, 18]. More recently, a confirmatory phase II, randomized, double-blind, placebo-controlled trial in HIV-infected, anti-Tat antibody negative, antiretroviral-treated adult volunteers [18], (ISS T-003, *ClinicalTrial.gov identifier: NCT01513135*) has been completed in South Africa, an endemic region in which approximately 28 % of individuals living with HIV and tuberculosis (TB) reside [19]. Infections caused by *Mtb* are the main and most dangerous co-infections in HIV/AIDS patients. It is estimated that one-third to one-half of the over 30 million AIDS death can be ascribed to TB. Especially in the endemic regions, *Mtb* and HIV co-infection hampers control of both diseases. Thus, it is of relevance to verify whether vaccines or immunotherapies against HIV infections can be safely administered to individuals infected by *Mtb*. In fact, the lack of this information has prevented enrollment of HIV-1 infected individuals with active TB in the Tat vaccine therapeutic trial conducted in South Africa [18], (ISS T-003, *ClinicalTrial.gov identifier: NCT01513135*).

Thus, to start addressing the interplay and reciprocal effect of Tat vaccination on *Mtb* infection, we investigated the effects of Tat vaccination on the outcome of active *Mtb* infection and on the protective efficacy of Bacillus Calmette-Guerin (BCG), the current TB vaccine, in a murine TB model. The immunogenicity of the Tat vaccine in these contexts was also assessed.

## Methods

### Microorganims

*M. tuberculosis* H37Rv (ATCC 27294) and *M. bovis* BCG strain Pasteur (ATCC 27291) were grown at 37 °C in Middlebrook 7H9 medium supplemented with albumin-destrose-catalase enrichment, under agitation (120 rpm), up to mid-exponential phase. Aliquoted stocks were stored at −70 °C until use.

### Reagents

HIV-1 Tat protein from IIIB-BH-10 (subtype B) strain was produced in *Escherichia coli* and prepared as previously reported [20]. The lipopolysaccharide content of this preparation was measured by a *Lymulus* amebocyte lysate test and shown to be <0.06 EU/μg of protein. The recombinant (r)Ag85B protein was prepared as previously reported [21]. The lipopolysaccharide content of this preparation was measured by a *Lymulus* amebocyte lysate test and shown to be below 4.3 EU/μg of protein. All these reagents were purchased from Diatheva, (Fano, PU, Italy). Purified protein derivative (PPD) was purchased from Statens Serum Institute*,* (Copenhagen*,* Denmark).

### Experimental design

**Fig. 1:**
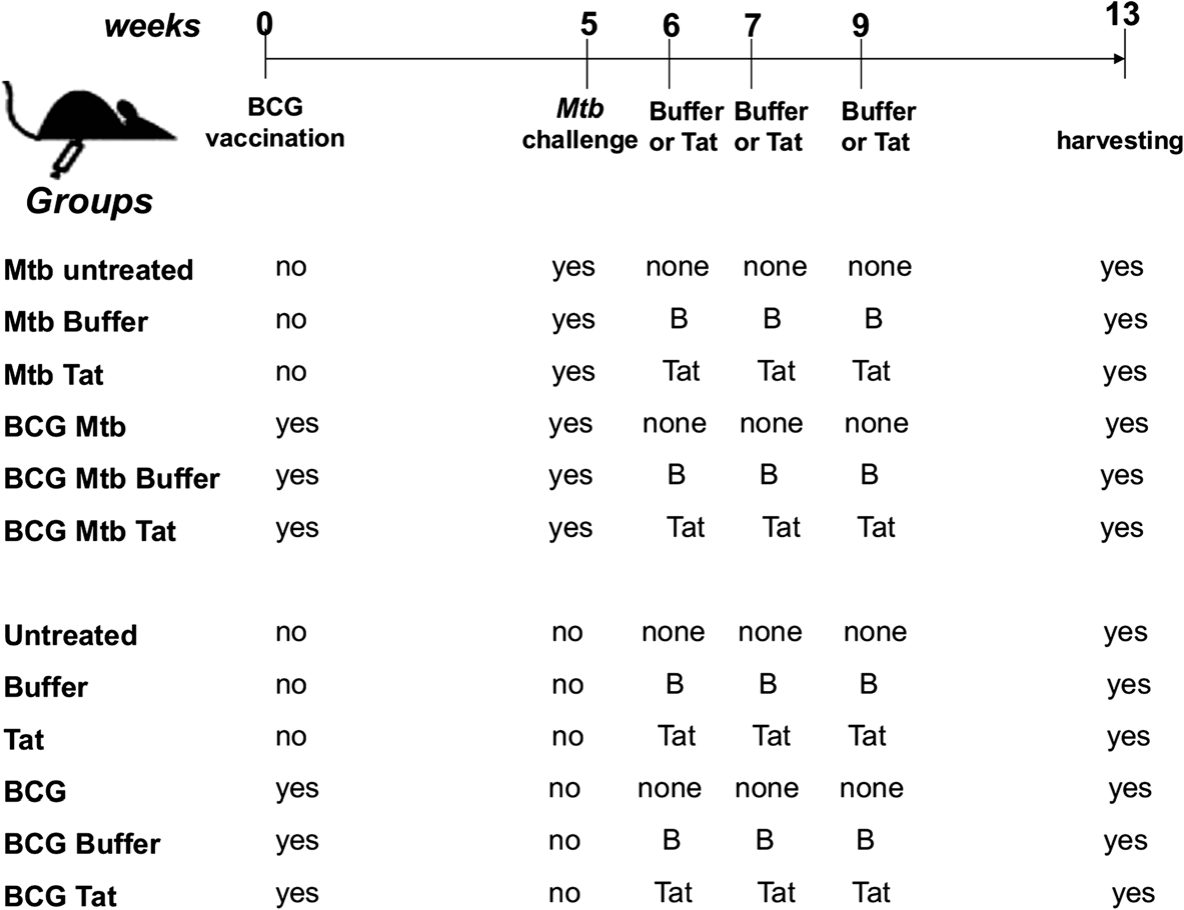
Schematic diagram of the experimental design. C57BL/6 female mice (6 mice/groups) were vaccinated subcutaneously with a single dose of BCG Pasteur (10^5^ CFU). Five weeks later, BCG-vaccinated and unvaccinated mice were challenged i.v. with 10^5^ CFU of *Mtb* H37Rv. The Tat protein (10 μg/mouse) in albumin buffer, or albumin buffer only were administered intradermally at 1, 2 and 4 weeks post *Mtb* infection. As control, some mice (5 mice/groups) were either vaccinated or not with BCG, were not challenged with *Mtb* (*Mtb*-free mice)*,* but received the Tat protein or albumin buffer with a schedule identical to that of *Mtb*-infected mice. At 8 weeks post *Mtb* infection and 13 weeks post BCG vaccination, all mice were killed and the *Mtb*-related pathology and immune response against *Mtb* antigens or Tat protein were evaluated

C57BL/6 female mice were supplied as specific pathogen-free mice by Charles River (Calco, Lecco, Italy) and were maintained in specific-pathogen-free conditions. Food and water were available ad libitum. According to the experimental design drawn in Fig. 1, 4 weeks old mice were immunized with a single dose of BCG (10^5^ CFU) injected subcutaneously. After five weeks, BCG-vaccinated and unvaccinated mice were challenged intravenously (i.v.) in a lateral tail vein with 10^5^ CFU of *Mtb* H37Rv. Infection studies were performed in a biosafety level 3 facility; mice were housed in microisolator cages and fed with autoclaved food and water at libitum. Tat protein (10 μg/100 μL of a buffer containing 0.5 % albumin) was injected intradermally at 1, 2 and 5 weeks after the *Mtb* challenge. Mice treated only with buffer served as control. BCG and Tat protein were also administered, with identical timing, to *Mtb*-free mice, as controls. Thirteen weeks after BCG vaccination, corresponding to 8 weeks post *Mtb*-infection and 4 weeks after the last Tat administration, all uninfected and *Mtb*-infected mice were sacrificed by cervical dislocation, according to the ethics requirements. The degree of pathology and levels of specific anti-*Mtb* or anti-Tat immune responses were evaluated. Five or six mice were used for each experimental mouse group. In total we used 66 mice.

### Histology and morphometry

All right lung lobes from each mouse were fixed in buffered formalin and subsequently embedded in paraffin; the tissue was then cut in 5-μm sections. For morphometry, 5-μm sections of entire paraffin-embedded lung lobes were stained with hematoxylin-eosin, were magnified at 2 and 40× by use of a Leica DM LB microscope, and were photographed by an HV-C20A camera (Hitachi). The histopathological parameters were evaluated and granuloma formation was scored by estimating the area occupied in the lung section. Appropriate software (Scion Image; Scion) was used to measure both the total and the infiltrated area across the whole lobe of the lung. All sections were evaluated in a blinded fashion by 2 investigators.

### Lung homogenates

Lung homogenates were prepared by mechanical homogenization of the left lobe of lungs in distilled water containing 0.01 % Tween 80 and protease inhibitors (complete Mini, Roche Diagnostic, Indianapolis, IN, USA) using a Gentle Macs dissociator (Miltenyi Biotec Srl, Calderara di Reno, Italy). Lung homogenates were used freshly for CFU assay; the homogenate was spun free from any debris by centrifugation and the supernatant filtered before being assayed for cytokine detection.

### Spleen cell preparation

Single cell suspension from spleens were prepared in 2 ml of PBS by mechanic dissociation (Gentle Macs dissociator); 200 μl were used freshly for CFU assay, the left was applied to Falcon 2360 cell strainers (BD Discovery Labware, San Diego, CA), centrifuged, separated into aliquots and frozen for use in immunological assays.

### CFU assay

The number of bacteria in lung homogenates or in spleen cell suspensions after lysis with saponin 0.1 % in distilled water was enumerated by plating 10-fold dilutions, prepared in distilled water, on Middlebrook 7H10 agar. The colonies were counted visually after 21 days of incubation.

### Cell cultures

Spleen cells from both uninfected and *Mtb*-infected mice were cultured in 96-well plates (4x10^5^ cells/well) in RPMI-1640 supplemented with 10 % heat-inactivated FBS, 2 mM L-glutamine, 10 mM HEPES buffer, 50 μM 2-mercaptoethanol. Fifty U/ml penicillin and 50 μg/ml streptomycin were added to cultures of cells from uninfected mice. Cells were stimulated with 5 μg/ml rAg85B protein, 2 μg/ml PPD for 96 h, before detection of Interferon (IFN)-γ and Interleukin (IL)-17 production in culture supernatants.

### Cytokine detection

Supernatants of spleen cell cultures and lung homogenates were assayed for cytokine/chemokines detection by quantitative sandwich ELISA specific for IFN-γ, IL-17, IL-1β, IL-22 and Chemokine (C-C motif) ligand-4 (CCL-4) (mouse Quantikine, R&D System, Inc.), in accordance with manufacturer’s instructions.

### Antibody measurement

The levels of total anti-Tat IgG antibodies in the sera collected by retro-orbital bleeding were determined by ELISA, as previously described [22]. Briefly, polyvinyl microtiter plates (Nunc) were coated overnight at 4 °C with 100 ng of Tat/well in 200 μL of 0.05 mol/L carbonate buffer (pH 9.6). Plates were blocked with PBS containing 1 % bovine serum albumin (BSA) and 0.05 % Tween 20 or milk 1 % and several dilutions of mouse sera were then incubated for 90 min at 37 °C. Horseradish peroxidase-conjugated goat μ-Mouse IgG (Sigma) and then ABTS solution (Roche Diagnostic SpA) were used for anti-Tat IgG detection. The mean absorbance of 1/100-diluted, naïve, uninfected mouse sera plus 3 standard deviations was adopted as the cutoff absorbance for determining antibody titers.

### Statistical analysis

GraphPad Prism 6 Software was used to perform one-way or two-way ANOVA and Tukey’s multiple comparisons post-test. A *P*-value of < 0.05 was considered significant.

## Results

### Tat protein vaccination is well tolerated in both *Mtb*-infected and *Mtb*-free mice

Mice were infected with virulent *Mtb* by i.v. route 5 weeks after BCG vaccination. Tat protein was injected intradermally at 1, 2 and 4 weeks after *Mtb* challenge with a protocol of administration resembling that used in the clinical trials [12–15]. The scheme of the experimental design is illustrated in Fig. 1.

Vaccination with Tat protein was well tolerated in both *Mtb*-infected and *Mtb*-free mice, irrespective of BCG vaccination. No evidence of toxicity, as judged by mortality, body weight, food consumption, behavioral and gross examination of vital organs at the time of sacrifice was observed in Tat-treated *Mtb*-free mice (data not shown). In *Mtb*-infected mice, all those parameters were comparable among Tat-treated, buffer-treated and untreated groups (data not shown).

### Administration of Tat protein to *Mtb*-infected mice reduces lung immunopathology without affecting bacterial load

**Fig. 2:**
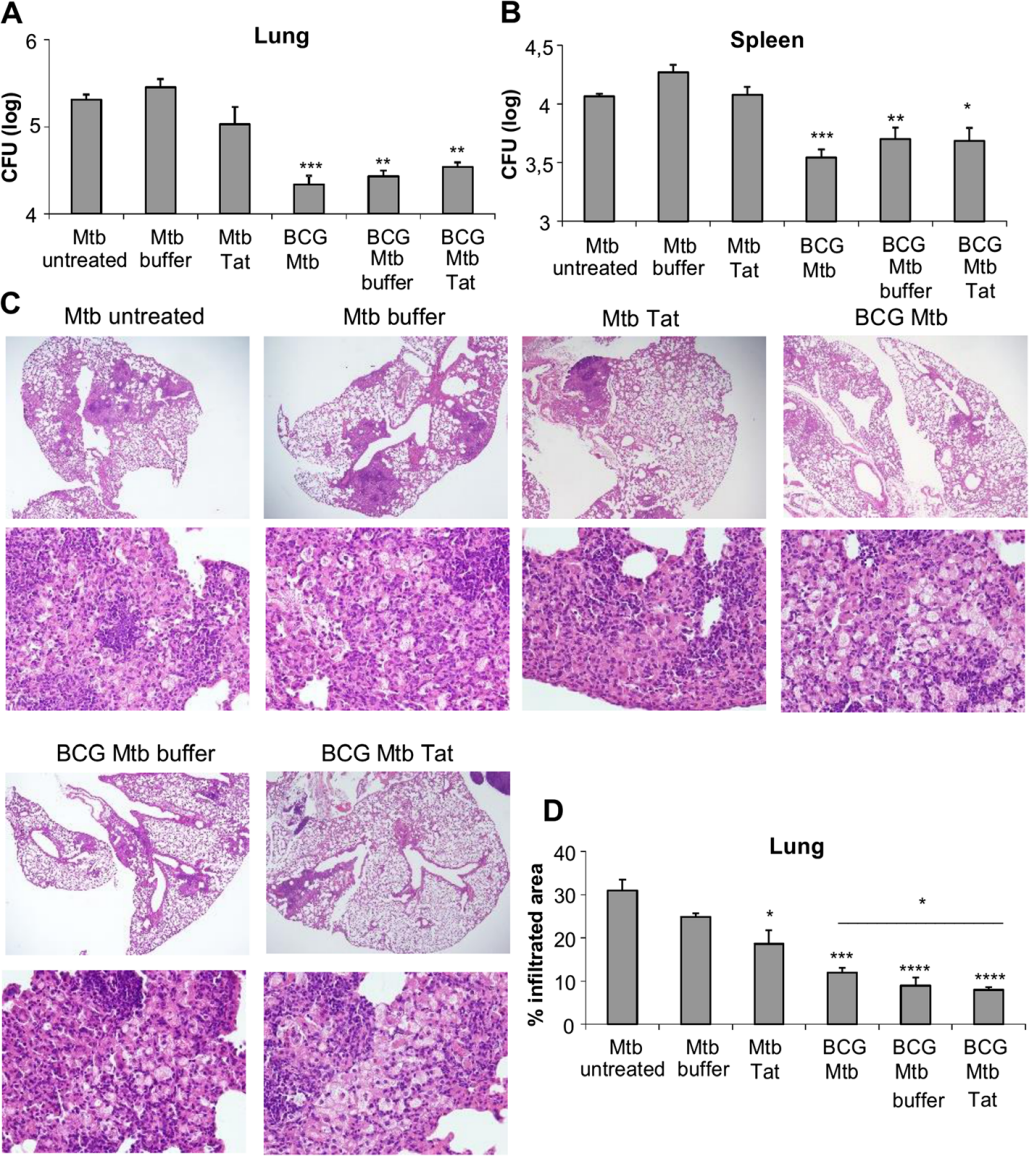
Tat vaccine administered to *Mtb*-infected mice reduces pulmonary infiltration and does not abrogate the protection conferred by BCG vaccination. Eight weeks post *Mtb* infection, bacteria in the lung (**a**) and spleen (**b**) were enumerated by CFU assay in mice treated as shown in Fig. 1. **c** Comparison of percentages of infiltrated area of sections of lung right lobes from the different mice groups. All data are expressed as mean of 6 individual mice/group ± SE. Statistical analysis was done by one-way ANOVA and Tukey’s multiple comparisons post-test *, *P* < 0.05, **, *P* < 0.01, ***, *P* < 0.001, differences respect to untreated *Mtb*-infected group. In panel C, the difference between BCG and BCG Tat-treated group is indicated. **d** Hematoxylin-eosin–stained sections of paraffin-embedded lungs from *Mtb*-infected mice killed at the 8 weeks post infection (upper panel) (magnification, ×2) and *(*lower panel) (magnification, ×40)

Tat vaccination did not affect the bacterial load in both lungs and spleens of *Mtb*-infected mice (Fig. 2a, b). The Tat-treated group showed only a slight and non-significant reduction in CFU levels compared to untreated or buffer-treated, *Mtb-*infected mice, (Fig. 2a, b). Of note, administration of Tat protein reduced *Mtb*-driven lung immunopathology. When lung sections were examined microscopically, untreated mice were found to develop larger areas of inflammatory infiltration than Tat-treated mice (Fig. 2c); in particular, the cellular infiltrates in the total area of lung sections, which was 31 ± 2.6 % in untreated mice, almost halved (18.7 ± 3.2 %) in Tat-treated mice (Fig. 2d). Lungs of *Mtb-*infected mice, irrespective of Tat-treatment showed a nodular and diffuse infiltration in the peribronchial, perivascular and septal interstitial space, with the infiltration also present in the alveolar spaces. Also the cell composition of granulomatous lesions was similar among the groups of *Mtb*-infected mice. It consisted mainly (60-70 %) of epitheliod macrophages sometimes with eosinophilic cytoplasm, sometimes with clear vesicular degeneration and foamy appearance (Fig. 2c), and the remaining 30-40 % represented by lymphocytes and rare neutrophils. No necrotic core or destruction of the lung parenchima was present.

**Fig. 3:**
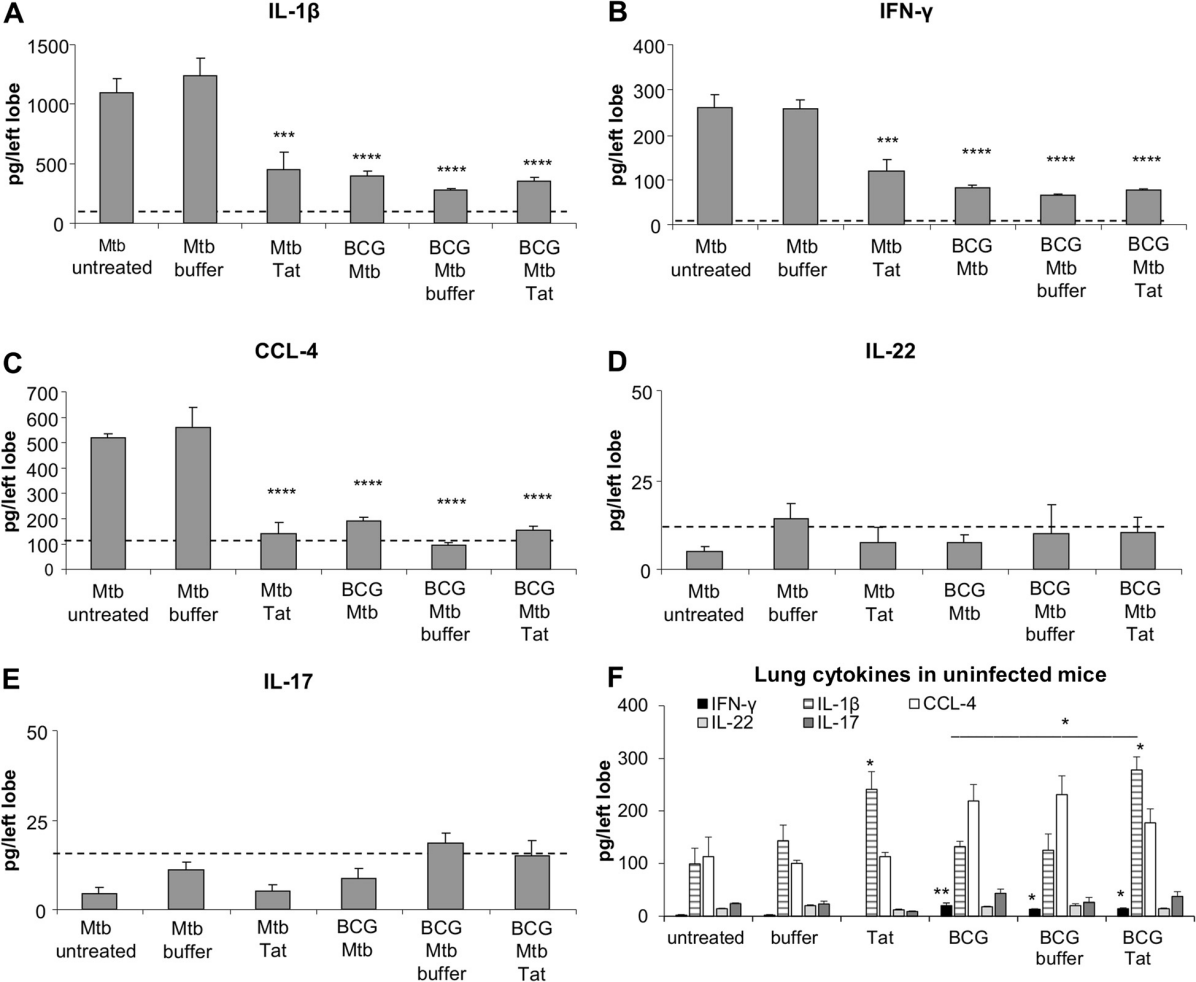
Lung protein levels of IFN-γ, CCL-4 and IL-1β are lowered in Tat-vaccinated, *Mtb*-infected mice. Left lungs of *Mtb*-infected (**a**-**e**) or uninfected (**f**) mice treated as reported in Fig. 1 were homogenized and then assayed for detection of the indicated cytokine/chemokine by commercial ELISA kits. In plots A-E, the dotted line represent the mean protein levels of the specific cytokine/chemokine detected in lung homogenate of untreated *Mtb*-free mice. All data are expressed as mean of 6 or 5 individual mice/group ± SE. Statistical analysis was done by one-way ANOVA and Tukey’s multiple comparisons post-test *, *P* < 0.05, **, *P* < 0.01, ***, *P* < 0.001, differences respect to untreated *Mtb*-infected group (**a**-**e**) or to untreated *Mtb*-free group (**f**). In panel F, the differences between BCG Tat-treated group versus both BCG and BCG buffer groups are indicated

In agreement with histological examination, Tat vaccination lowered the protein levels of IFN-γ, CCL-4 and IL-1β, in the lungs of *Mtb*-infected mice (Fig. 3a-c). Elevated pulmonary amounts of these *Mtb*-induced cytokines/chemokines correlate with a more severe outcome during the chronic phase of *Mtb* infection [23, 24]. In contrast, *Mtb* infection did not induce expression of IL-17 and IL-22 whose low levels remained unaltered in the lungs upon Tat administration (Fig. 3d, e).

### Protection against *Mtb* infection conferred by the BCG vaccine remains good in mice receiving Tat protein vaccine

We evaluated whether the administration of Tat vaccine could alter the protection conferred by BCG, the gold standard TB vaccine. Respect to unvaccinated mice, BCG vaccination significantly reduced the bacterial load in both lungs and spleen (Fig. 2a, b), decreased areas of inflammatory infiltration (Fig. 2c, d), and lowered the lung levels of IFN-γ, IL-1β and CCL-4 (Fig. 3a-c). The BCG-mediated protective effects were maintained in mice receiving Tat vaccination post *Mtb*-infection. No significant differences were observed among the three BCG-vaccinated, *Mtb*-infected groups in terms of bacterial loads (Fig. 2a, b) and cytokine/chemokine expression in the lungs (Fig. 3a-e). Of note, administration of Tat protein further reduced the area of inflammatory infiltration, suggesting a beneficial effect on lung pathology development (Fig. 2c, d).

### Administration of Tat vaccine enhances the level of IL-1β in the lungs of both unvaccinated and BCG-vaccinated, *Mtb*-free mice

Lungs of Tat-treated, *Mtb*-free mice showed a normal tissue architecture and the absence of notable inflammatory infiltrates by histological examination (data not shown). The protein levels of IFN-γ, CCL-4, IL-17 and IL-22 were comparable among Tat-treated, buffer-treated and untreated, *Mtb*-free mice (Fig. 3f). Instead, the expression of IL-1β, a pro-inflammatory cytokine contributing to host immune defense against *Mtb* [23, 25], was significantly up-regulated by Tat vaccine administration.

In the lungs of *Mtb*-free mice, BCG vaccination induced the expression of IFN-γ (Fig. 3f) and the appearance of small inflammatory infiltrates consisting of lymphocytes (the prevalent cell type) and macrophages (data not shown). Tat vaccination did not affect the expression of IFN-γ and the mild inflammation triggered by BCG, while increased the protein level of IL-1β (Fig. 3f), as found in unvaccinated, *Mtb*-free mice (Fig. 3f).

### Administration of Tat vaccine enhances mycobacterial-specific IFN-γ and IL-17 responses in mice infected with *Mtb* and in *Mtb*-free mice vaccinated with BCG

**Fig. 4:**
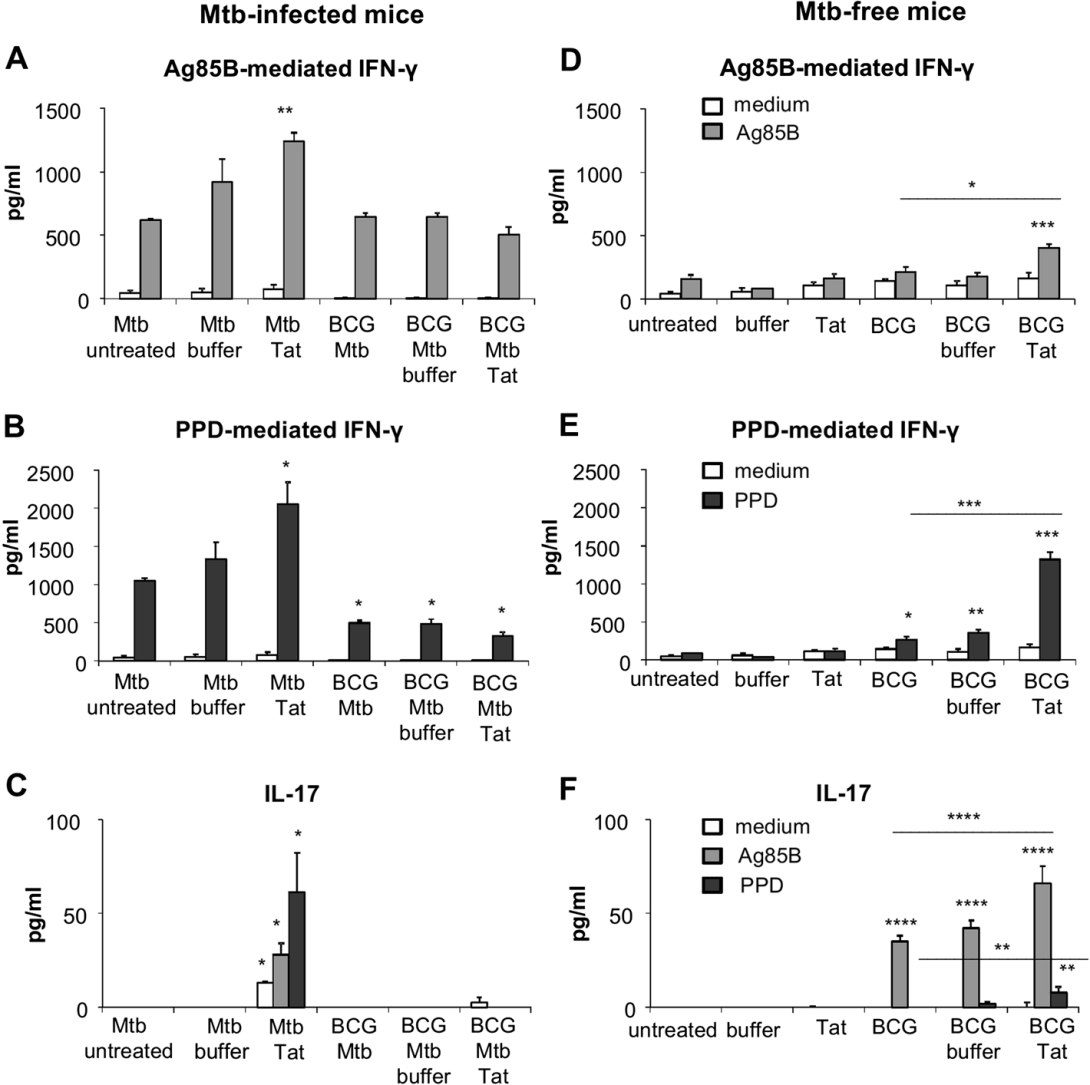
Tat vaccine enhances systemic IFN-γ and IL-17 responses specific for mycobacterial antigens in both *Mtb*-infected and BCG-vaccinated, *Mtb*-free mice. Spleen cells of *Mtb*-infected (**a**-**c**) or *Mtb*-free (**d**-**f**) mice treated as shown in Fig. 1 were stimulated *ex vivo* with 5 μg/ml of rAg85B protein or 2 μg/ml of PPD for 96 h. Culture supernatants were assayed for production of IFN-γ (**a**, **d**, Ag85B stimulation; **b**, **e**, PPD-stimulation) or IL-17 (**c**, **f**) by commercial ELISA kits. Each data point represents a pool of 5 or 6 mice, assessed in technical duplicates or triplicates and represents the mean ± SEM. The results shown are representative of two independent experiments. Statistical analysis was done by two-way ANOVA and Tukey’s multiple-comparison post-test*, *P* < 0.05, **, *P* < 0.01, ***, *P* < 0.001, ****, *P* < 0.0001, differences respect to untreated *Mtb*-infected group (**a**-**c**) or to untreated *Mtb*-free group (**d**-**f**). In panels **d**-**f**, the differences between BCG Tat-treated group versus both BCG and BCG buffer groups are indicated

We evaluated the effect of Tat vaccination on the development of systemic *Mtb*-specific immune responses, focusing on IFN-γ, which is essential for control of *Mtb* infection [26, 27] and IL-17, which appears to play a protective role in the early phase of *Mtb* infection [28]. Spleen cells recovered from both *Mtb*-infected and *Mtb*-free mice were stimulated *ex vivo* with Ag85B, an immunodominant antigen with a role in TB protection [21, 29–31], or PPD, the mixture of antigens released by *Mtb* during in vitro growth. Splenocytes of all groups of mice infected with *Mtb* produced significant amounts of IFN-γ, but not IL-17, in response to both Ag85B and PPD stimulation (Fig. 4a, b). Tat vaccine administration to *Mtb*-infected mice enhanced antigen-specific IFN-γ responses triggered by infection and even induced the production of IL-17 (Fig. 4a-c). However, the Tat-mediated immunomodulatory effects were not observed in BCG-vaccinated, *Mtb*-infected mice (Fig. 4a-c). Irrespective of Tat administration, no IFN-γ or IL-17 responses specific for mycobacterial antigens were detected in unvaccinated *Mtb*-free mice (Fig 4d-f). Instead, Tat protein administration potentiated the low mycobacterial-specific IFN-γ and IL-17 responses elicited by BCG vaccination in *Mtb*-free mice (Fig. 4d-f).

### The infection with *Mtb* reduces but does not suppress the production of anti-Tat antibodies which even is increased by BCG-vaccination in *Mtb*-free mice

**Fig. 5:**
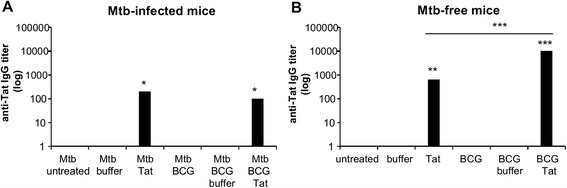
Tat vaccine administered to *Mtb*-infected mice is immunogenic. Serum samples of *Mtb*-infected (**a**) or *Mtb*-free (**b**) mice treated as shown in Fig. 1 were analyzed by ELISA for the presence of anti-Tat antibodies. Data are plotted as geometric mean ELISA titer ± SEM of 5 or 6 individual mice/group. The results shown are representative of two independent experiments. Statistical analysis was done by one-way ANOVA and Tukey’s multiple comparisons post-test *, *P* < 0.05, **, *P* < 0.01, ***, *P* < 0.001, differences respect to untreated *Mtb*-infected group (**a**) or to untreated *Mtb*-free group (**b**). In panel **b**, the difference between BCG-vaccinated and unvaccinated, Tat-treated groups is indicated

Finally, we determined whether mice infected with *Mtb* and vaccinated with Tat developed anti-Tat antibodies, a key parameter to evaluate the immunogenicity of the Tat vaccine and a *bona fide* correlate of efficacy [8, 14, 18, 32]. Mice receiving Tat vaccine post-*Mtb* infection were still able to mount a specific humoral response against Tat (Fig. 5a). The titers of anti-Tat IgG detected in sera of Tat-treated, infected mice (Fig. 5a), were only slightly lower than those measured in Tat-treated, *Mtb*-free mice (Fig. 5b). In Tat-treated, *Mtb*-infected mice, BCG vaccination did not affect the production of anti-Tat antibodies (Fig. 5a). On the other hand, in *Mtb*-free mice, a BCG priming before administration of Tat vaccine (the BCG vaccinated, Tat-treated group) potentiated the production of IgG specific for Tat respect to unvaccinated, Tat-treated mice (Fig. 5b).

## Discussion

The HIV-1 Tat protein-based vaccine, a promising therapeutic vaccine for AIDS patients, is in advanced clinical development [18, 19], (ISS T-003, *ClinicalTrial.gov identifier: NCT01513135*). Here we show that the administration of Tat vaccine to mice infected with *Mtb*, the main coinfection of AIDS, was well-tolerated, immunogenic, did not abrogate the protective efficacy of BCG, the current TB vaccine, and, overall, reduced the lung immunopathology caused by *Mtb.*

Tat protein vaccine did not affect bacterial growth in mice infected with *Mtb*. The scarce influence of Tat vaccine on bacterial load was in line with the results obtained in mice primed at 7 weeks post-*Mtb* infection with a MultiHIV DNA/protein vaccine encoding several HIV-1 products, including Tat [33] and with the outcome of *Mtb* H37Rv infection of Tat-transgenic mice, showing a lung bacterial burden comparable to that found in non-transgenic littermates [34]. However, *Mtb* growth is just one of the events that determine TB pathology. The immunocompetent C57BL/6 mice used in this study are able to contain *Mtb* replication in the lungs upon the initial exponential *Mtb* growth but cannot control the immuno-mediated lung pathology, which progressively worsens and causes death [35]. In this context, the *Mtb*-triggered expression of IFN-γ, IL-1β and CCL-4 in the lungs [23] plays a dual, opposite role. In the early stage of infection, these pro-inflammatory cytokines/chemokines trigger protective mechanisms [23], while their persistence in the late stages, indicates the inability of controlling/clearing the infection and represents a major marker of progressing TB [24]. Unexpectedly, we found that Tat protein vaccination, when compared to untreated mice, limited *Mtb* infection-driven lung immunopathology by reducing both the protein levels of IFN-γ, IL-1β and CCL-4 and the area of cellular infiltration. When the *Mtb*/Tat-treated group was compared to the *Mtb*/buffer group differences were less evident with regard to the infiltrated area, while differences remained significant with respect to the reduction of cytokine/chemokine levels, confirming the ability of Tat vaccine to improve lung immunopathology.

Since containment of lung damage occurred independently from bacterial load control, it is conceivable that Tat-treatment affected immune/inflammatory mechanisms driven by the infection but not closely related to *Mtb* active replication. Of note, incapacity to control progression of lung disease due to an unstoppable inflammation despite containment of *Mtb* load is a key feature of TB pathology in these mice [35]. We know little about the sustainability and mechanisms involved in Tat mediated-improvement of lung pathology. However, the Tat-mediated protective/immunosuppressive effects persisted up to study completion, that is 4 weeks after the last Tat dose, and were independent of IL-17/IL-22 axis and lung neutrophil influx, which cause the high susceptibility to *Mtb* infection in mice [36]. The containment of excessive *Mtb*-triggered inflammatory responses is a novel, and thus far unreported, feature of Tat-mediated immune-modulatory capabilities, which may have important implications in public health. Control of excessive inflammation to prevent lung damage is relevant in human pulmonary TB [37] and correlates with a better prognosis and efficacy of chemotherapy in TB patients [38].

In sharp contrast with our findings, Tat has been reported to favor TB progression [34]. In particular, an accelerated mortality rate, poorly organized lung granuloma-lesions and increased IFN-γ and TNF-α expression as signs of the worsening outcome have been reported in Tat transgenic mice [34]. However, differences between our model of Tat protein vaccination and the Tat transgenic mouse model are substantial, and may explain the discrepancy of results. In Tat-transgenic mice, Tat is permanently expressed in large amounts in all tissues, and can be found both intra- and extra-cellularly. More importantly, Tat-transgenic mice are tolerant to Tat and do not mount an anti-Tat immune response. To the opposite, in our vaccine model tiny amounts of Tat protein were administered a few times in mice, which mounted an antibody response to the protein. Thus, in our model, exposure to Tat was much lower and shorter, and further reduced by the ensuing antibody response, as compared to Honda's transgenic one [34].

In Tat-vaccinated, *Mtb*-infected mice, the reduction of both bacterial load and lung immunopathology conferred by BCG was maintained, indicating that Tat treatment did not abrogate the vaccine efficacy of BCG. Our data provide preliminary evidence for the use of Tat vaccine in those countries with high HIV/TB co-endemicity and where BCG vaccination is mandatory [39]. BCG, given to over 3 billion individuals, in the setting of routine newborn immunization in high endemic TB countries, is effective in controlling the severe forms of pediatric TB [39], although unable to control pulmonary TB in adult patients [40].

Tat vaccination in *Mtb*-infected mice potentiated systemic IFN-γ and IL-17 responses specific for mycobacterial antigens, including Ag85B. The ability of Tat to activate antigen unrelated Th1 responses is well known, and several of the involved mechanisms have been described [20, 41–44]. Tat protein alters lymphocyte signaling (e.g. activation of NF-kB), modulates dendritic cell maturation, enhances antigen processing and presentation and promotes the induction and effector functions of memory T cells [20, 41–44]. Instead, little it is known about the Tat-mediated modulation of IL-17 response. Recently, it has been reported that Tat increases IL-17 production by T cells, via activation of the vascular endothelial growth factor receptor 2 [45]. Since Ag85B-specific CD4^+^ T cells are induced to produce IL-17 through antigen-unrelated activation of dendritic cells, particularly of the CD8α^−^ subtype [46], we cannot exclude that, in our model, Tat promoted IL-17 response via its known ability to activate dendritic cells [20, 41]. Whether the Tat-mediated modulation of mycobacterial-specific IFN-γ and IL-17 responses might have contributed to lowering lung immunopathology remains to be investigated. It is known that protection from *Mtb* infection is dependent on robust IFN-γ secretion by antigen-specific CD4^+^ T cells [26] and, to a lesser extent, on Th17 responses [28]. In particular, activation of IFN-γ response specific for Ag85B, a leading TB vaccine candidate [29], (http://www.*clinicaltrials.gov/show/NCT01049282*), results in a better control of *Mtb* infection in numerous experimental animal models [21, 30, 31].

Curiously, the Tat-mediated enhancement of immune responses was observed in unvaccinated, *Mtb*-infected mice and BCG-vaccinated uninfected mice, but not in BCG-vaccinated, *Mtb*-infected mice. This finding was unexpected, and we do not have a definitive explanation for it. It is conceivable that this latter condition, a recall response to BCG triggered by an acute *Mtb* infection, markedly differs, with respect to either BCG vaccination (untriggered memory) or *Mtb* infection alone (primary response), in terms of antigen load, inflammatory environment and quality of adaptive and/or innate immune responses.

In addition to the modulation of the adaptive response, Tat may improve the control of *Mtb*-mediated lung immunopathology by affecting innate immunity. In the lungs of *Mtb*-free animals vaccinated with Tat, a significant increase of IL-1β was found 4 weeks after the last Tat dose. Interleukin-1β is important for host immune defense against *Mtb* [23, 25], likely increasing the mycobactericidal activity of macrophages [47], the main cell type harboring the pathogen.

Finally, we evaluated the impact of *Mtb* infection and/or BCG vaccination on the development of anti-Tat antibodies, a key parameter to evaluate immunogenicity of the Tat vaccine and a *bona fide* correlate of HIV vaccine efficacy [8, 14, 18, 32]. *Mtb* infection only slightly reduced the production of specific anti-Tat IgG. Similar results were observed in *Mtb*-infected mice vaccinated with a MultiHIV DNA/protein vaccine, which included Tat [33]. The moderately high titer of anti-Tat serum IgG elicited by this vaccine was not altered by the concurrent *Mtb* infection, despite an impairment in both the magnitude and quality of antibodies against other vaccine components (p24 Gag and gp160 Env) [33]. Altogether, these data indicate that *Mtb*-infected hosts are still able to mount a humoral response against the Tat vaccine, a mandatory step for vaccine efficacy. However, it remains to be determined whether the anti-Tat antibodies elicited in the presence of *Mtb*-infection are effective at neutralizing the biological activities of Tat, as required for Tat vaccine efficacy [14, 32].

In Tat-vaccinated, *Mtb*-free mice, a priming with BCG increased the production of anti-Tat antibodies. Likely, this effect was due to the “adjuvant” capabilities of this live non-pathogenic vaccine. Indeed, BCG has been used to treat non-muscle-invasive bladder cancer for more than 30 years [48], being one of the most successful biotherapies for cancer in use [49]. BCG triggers the secretion of pro-inflammatory cytokines [50], modulates the activation of granulocytes, macrophages, and dendritic cells [51, 52] and promotes bystander activation of CD4^+^ and CD8^+^ lymphocytes [53]. In line with those findings, we observed signs of a very mild inflammation in the lungs of BCG vaccinated, *Mtb*-free mice, highlighted by the presence of small foci of infiltrates and by the expression of IFN-γ as late as 13 weeks after the subcutaneous BCG inoculum. The concurrent *Mtb* infection subverted the BCG-mediated increase of anti-Tat antibodies. It is conceivable that the inflammatory/immune responses triggered by the virulent *Mtb*, and mitigated only in part by BCG vaccination, were predominant, and abrogated the BCG-mediated “adjuvant” effects.

Although the route of *Mtb* infection used in our studies is unnatural, reports from several studies indicate that *Mtb*-driven pulmonary pathological events, such as granuloma formation and expression of cytokines/chemokines as well as vaccine efficacy, are comparable in mice infected with *Mtb* either by i.v. or by aerosol route [54–56]. Accordingly, although confirmation of our results in mice challenged by the aerosol route is warranted, we are confident that the i.v. route did not invalidate the significance of our findings. Moreover, given the high proportion of individuals with active TB in countries where HIV-1 is highly prevalent, it will be of importance to assess in future studies whether Tat vaccination is beneficial also during anti-TB chemotherapy.

## Conclusions

The anti-HIV Tat protein vaccination administered to mice infected i.v. with *Mtb*: 1) did not favour *Mtb* replication; 2) limited the pathogen-driven immuno-mediated lung injury for at least 4 weeks after the last Tat dose; 3) did not abrogate the protective efficacy of BCG, the current TB vaccine; 4) enhanced early systemic Mtb-specific IFN-γ responses, essential for control of *Mtb* infection; 5) was immunogenic, as indicated by the production of anti-Tat antibodies; 6) was well tolerated. In addition, BCG vaccination enhanced the anti-Tat specific humoral response in Tat-vaccinated, *Mtb*-free mice. Our findings provide preliminary evidence of the feasibility, in terms of safety and immunogenicity, of administering the Tat vaccine in BCG-vaccinated and/or *Mtb*-infected subjects. These results are of clinical relevance considering that people who mostly need vaccines against HIV/AIDS live in geographical areas where TB is highly prevalent.

## Abbreviations

BCG, Bacillus Calmette-Guerin; CCL-4, Chemokine (C-C motif) ligand-4; i.v., intravenous; IFN, Interferon; Ig, immunoglobulin; IL, Interleukin; *Mtb, Mycobacterium tuberculosis*; PPD, purified protein derivative; TB, tuberculosis
